# Characteristics of T-cell large granular lymphocyte proliferations associated with neutropenia and inflammatory arthropathy

**DOI:** 10.1186/ar2424

**Published:** 2008-05-12

**Authors:** Monika Prochorec-Sobieszek, Grzegorz Rymkiewicz, Hanna Makuch-Łasica, Mirosław Majewski, Katarzyna Michalak, Robert Rupiński, Krzysztof Warzocha, Renata Maryniak

**Affiliations:** 1 Department of Pathomorphology, Institute of Hematology and Transfusion Medicine, I. Gandhi 14, 02-776 Warsaw, Poland; 2 Department of Pathology, Institute of Rheumatology, Spartańska 1, 02-637 Warsaw, Poland; 3 Department of Pathology, The Maria Skłodowska-Curie Memorial Cancer Center and Institute of Oncology, Roentgena 5, 02-781 Warsaw, Poland; 4 Molecular Biology Laboratory, Institute of Hematology and Transfusion Medicine, I. Gandhi 14, 02-776 Warsaw, Poland; 5 Department of Internal Diseases and Hematology, Institute of Hematology and Transfusion Medicine, I. Gandhi 14, 02-776 Warsaw, Poland; 6 Department of Rheumatology, Institute of Rheumatology, Spartańska 1, 02-637 Warsaw, Poland; 7 Department of Hematology, Institute of Hematology and Transfusion Medicine, I. Gandhi 14, 02-776 Warsaw, Poland

## Abstract

**Introduction:**

The purpose of this study was to analyze the data of patients with T-cell large granular lymphocyte (T-LGL) lymphocytosis associated with inflammatory arthropathy or with no arthritis symptoms.

**Methods:**

Clinical, serological as well as histopathological, immuhistochemical, and flow cytometric evaluations of blood/bone marrow of 21 patients with T-LGL lymphocytosis were performed. The bone marrow samples were also investigated for T-cell receptor (*TCR*) and immunoglobulin (*IG*) gene rearrangements by polymerase chain reaction with heteroduplex analysis.

**Results:**

Neutropenia was observed in 21 patients, splenomegaly in 10, autoimmune diseases such as rheumatoid arthritis (RA) in 9, unclassified arthritis resembling RA in 2, and autoimmune thyroiditis in 5 patients. T-LGL leukemia was recognized in 19 cases. Features of Felty syndrome were observed in all RA patients, representing a spectrum of T-LGL proliferations from reactive polyclonal through transitional between reactive and monoclonal to T-LGL leukemia. Bone marrow trephines from T-LGL leukemia patients showed interstitial clusters and intrasinusoidal linear infiltrations of CD3^+^/CD8^+^/CD57^+^/granzyme B^+ ^lymphocytes, reactive lymphoid nodules, and decreased or normal granulocyte precursor count with left-shifted maturation. In three-color flow cytometry (FCM), T-LGL leukemia cells demonstrated CD2, CD3, and CD8 expression as well as a combination of CD16, CD56, or CD57. Abnormalities of other T-cell antigen expressions (especially CD5, CD7, and CD43) were also detected. In patients with polyclonal T-LGL lymphocytosis, T cells were dispersed in the bone marrow and the expression of pan-T-cell antigens in FCM was normal. Molecular studies revealed *TCRB *and *TCRG *gene rearrangements in 13 patients and *TCRB*, *TCRG*, and *TCRD *in 4 patients. The most frequently rearranged regions of variable genes were V_β_-J_β1_, J_β2 _and V_γ _If V_γ10_-J_γ_. Moreover, in 4 patients, additional rearrangements of *IG *kappa and lambda variable genes of B cells were also observed.

**Conclusion:**

RA and neutropenia patients represented a continuous spectrum of T-LGL proliferations, although monoclonal expansions were most frequently observed. The histopathological pattern and immunophenotype of bone marrow infiltration as well as molecular characteristics were similar in T-LGL leukemia patients with and without arthritis.

## Introduction

The etiology of such abnormalities as lymphocytosis, neutropenia, and arthropathy diagnosed either by a rheumatologist or a hematologist often remains obscure. These clinical findings may be associated with the presence of circulating T-cell large granular lymphocytes (T-LGLs) [1-3]. LGL disorders comprise a spectrum of polyclonal, oligoclonal, or monoclonal expansions [4], which arise mostly from mature, activated cytotoxic T lymphocytes (T-LGL) CD3^+^/CD8^+^/CD57^+^/CD16^+ ^and less often from natural killer cells (NK-LGL) CD3-/CD2^+^/CD56^+^/CD16^+ ^[5]. Clinically pronounced monoclonal proliferation of T-LGLs with bone marrow and spleen infiltration is diagnosed as T-LGL leukemia, a rare, indolent, chronic disorder with characteristic features such as mild lymphocytosis, neutropenia, and anemia. They may be autoimmune by nature or result from a T-cell-mediated suppressor effect on hemopoesis [6,7]. The T-LGL leukemia diagnosis is confirmed by monoclonal T-cell receptor (*TCR*) gene rearrangement detected in abnormal CD3^+^/CD57^+ ^cell populations [5,6]. An interesting feature of T-LGL leukemia is its strong association with a number of autoimmune disorders and immunological abnormalities, most common in patients with rheumatoid arthritis (RA) (30% of patients), which usually precedes or develops concurrently with the hematological process [8-10]. Patients with T-LGL leukemia and accompanying RA closely resemble patients with Felty syndrome (FS) in clinical presentation: neutropenia, RA, variable splenomegaly, and immunogenetic findings such as a high prevalence of HLA-DR4 [11,12]. Moreover, monoclonal T-LGL lymphocytosis may be found in up to one third of FS patients [11,13-15]. Burks and Loughran [7] suggest that these two entities represent variants of the same clinicopathologic process. The aim of the present study was to perform an extensive clinical, histopathological, flow cytometric as well as genetic evaluation of 21 patients with T-LGL lymphocytosis associated with inflammatory arthropathy or with no arthritis symptoms. Our results demonstrate that patients with RA and neutropenia represent a continuous spectrum of T-LGL proliferations although monoclonal expansions are observed most frequently. The histopathological pattern and immunophenotype of the bone marrow infiltration as well as molecular characteristics were similar in T-LGL leukemia patients with and without arthritis.

## Materials and methods

A group of 21 patients with lymphocytosis and neutropenia, including several with arthropathy and splenomegaly, was enrolled in this study. Written informed consent was obtained from all of the patients, and the study was approved by the local bioethical committee of the Institute of Hematology and Transfusion Medicine in Warsaw. Complete blood count with manual differential analysis of blood cells was performed in all cases. Blood smears stained with May-Grünwald-Giemsa were examined for the presence of large granular lymphocytes.

Features of articular disease were defined in terms of duration and diagnosis (American Rheumatism Association [ARA] criteria for diagnosis of RA) [16]. In some patients, tests were done for rheumatoid factor (RF) (nephelometry), anticyclic citrullinated peptide (CCP) antibodies and anticardiolipin antibodies (aCLs) (enzyme-linked immunosorbent assay, ELISA), antinuclear antibodies (ANAs) (Hep2 cells), and cytoplasmic and perinuclear antineutrophil cytoplasmic antibodies (ELISA), depending on the clinical presentation of the patient.

Trephine biopsies of all 21 patients were histopathologically examined. They were fixed in Oxford fixative, routinely processed, and stained with hematoxylin and eosin. Immunohistochemical studies were done (EnVision™ Detection Systems) (Dako Denmark A/S, Glostrup, Denmark) (DAKO) using the following mono- and polyclonal antibodies: CD3, myeloperoxydase, hemoglobin (polyclonal), CD20 (L26), CD8 (C8/144B) (DAKO) and CD4 (4B12), CD57 (NK-1), and granzyme B (11F1) (Novocastra, now part of Leica Microsystems, Wetzlar, Germany). Positive and negative controls were included.

Immunophenotyping of peripheral blood lymphocytes was performed in 15 patients with a three-color FACScalibur cytometer (flow cytometry, FCM) (Becton Dickinson, San Jose, CA, USA) (BD) and analyzed by CellQuest software (BD). Lymphocytes were treated with monoclonal antibodies against CD45 and HLA-DR; pan-B antigen: CD19 (BD); pan-T antigens: CD3 (DAKO), CD2, CD4, CD5, CD7, CD8, CD43, TCRαβ, and TCRγδ (BD); and NK-specific markers: CD16 (DAKO), CD56 (BD), CD57 (Sigma-Aldrich, St. Louis, MO, USA), and human IL-2 Rα receptor CD25 (BD). Isotype controls were used.

*TCR *genes as well as immunoglobulin heavy-chain (*IGH*) and kappa (*IGK*) and lambda (*IGL*) light-chain gene rearrangements were tested in 19 patients following the BIOMED-2 protocol [17]. DNA was isolated from blood/bone marrow mononuclear cells with the column method (Qiagen, Hilden, Germany) after Ficoll separation. *TCRBV-TCRJ *gene rearrangements were tested using 23 forward and 9 reverse primers (V_β_-J_β1_, J_β2_) and 23 forward and 4 reverse primers for regions V_β_-J_β2 _and 2 forward and 13 reverse primers for regions D_β1_, D_β2_-J_β_. *TCRG *gene rearrangements were tested using 2 forward and 2 reverse primers for regions V_γ _If, V_γ10_-J_γ _and 2 forward and 2 reverse primers for regions coding V_γ9_, V_γ11_-J_γ_. *TCRD *gene rearrangements were tested using 7 forward and 5 reverse primers for regions coding V_δ_, D_δ2_-J_δ_, D_δ3_. The *IGH *gene rearrangement test consisted of three multiplex polymerase chain reaction (PCR) tubes with 27 forward and 5 reverse primers, *IGK *tests consisted of 2 multiplex PCR tubes with 13 forward and 3 reverse primers, and the *IGL *test consisted of 1 multiplex PCR tube with 6 forward and 2 reverse primers. PCR products underwent heteroduplex analysis (95°C for 5 minutes and 4°C for 60 minutes) and were separated using electrophoresis on polyacrylamide gel and visualized by ethidium bromide. Cytogenetic studies on bone marrow aspirate samples of 7 patients were performed using a G-banding technique, and the results were analyzed according to International System for Human Cytogenetic Nomenclature (1995). The T-LGL leukemia diagnosis was made according to the World Health Organization (WHO) classification [6] in cases with monoclonal LGL lymphocytosis CD3^+^/CD57^+^/TCRαβ^+^/γδ^+ ^of more than 6 months in duration. Cases with circulating LGLs of greater than 2 × 10^9^/L in peripheral blood as well as patients with leucopenia and smaller expansions of LGL were included. The diagnosis of T-LGL lymphocytosis in 21 patients was based on blood and bone marrow tests, including immunophenotypic and molecular studies.

## Results

### Clinical and laboratory characteristics

The clinical symptoms and hematological data of 21 patients are summarized in Tables 1 and 2. The median age was 55.7 years (range 28 to 84 years). For all patients, the cell count detected in routine blood tests was abnormal. Lymphocytosis ranged from 0.8 to 34.5 × 10^9^/L and persisted for at least 6 months. On cytological examination of blood smears, lymphocytes consisted mainly of LGLs. Neutropenia (<1.5 × 10^9^/L) was the predominant hematological abnormality in 21 patients and was severe in 12 (<0.5 × 10^9^/L). Several patients had other cytopenias: leucopenia (white blood cells <4.5 × 10^9^/L) was diagnosed in 9 patients, anemia (hemoglobin <10 g/dL) in 5 patients, and thrombocytopenia (platelets <150 × 10^9^/L) in 9 patients.

**Table 1:** Basic clinical data, details of arthropathy, serologic findings, and therapy

Case	Age/gender	Clinical presentation and arthropathy	Spleen, mm	ANA	RF IgM, IU/mL	ANCA	CCP	aCL	HP	Therapy
1	84/F	RA (43 y), BCC (7 m), AITD, weight loss	120	1/160	423	Neg	Pos	Pos	Yes	Corticosteroids
2	56/F	RA (10 y), BCC (6 y), amyloidosis AA	115	1/160	320	Neg	Pos	ND	Yes	Corticosteroids
3	75/F	RA (7 y), BCC (2 y), weight loss	180	1/160	125	Neg	Pos	ND	Yes	Corticosteroids
4	36/F	RA (18 y), BCC (4 y), AITD	187	1/160	335	Neg	Pos	ND	Yes	Corticosteroids/MTX
5	58/F	RA (32 y), BCC (1 y), AITD	95	1/160	97	Neg	Pos	ND	Yes	Corticosteroids/MTX
6	74/M	RA (10 y), BCC (3 y)	150	1/320	325	Neg	Pos	Pos	Yes	Unknown
7	57/M	RA (10 y), BCC (3 y)	200	1/160	320	ND	Pos	ND	Yes	Corticosteroids/MTX
8	52/F	UA (12 y), BCC (3 y), recurrent infections, skin lesions	160	1/320	Neg	Neg	ND	Pos	Yes	Corticosteroids
9	68/F	BCC (17 y), UA (2 y), recurrent infections, skin lesions	185	1/80 dsDNA	97	Neg	Pos	Pos	Yes	G-CSF/MTX
10	51/F	RA (8 y), rheumatoid nodules, BCC (3 y)	185	1/160	450	Neg	Pos	Pos	Yes	MTX/CSA/Corticosteroids
11	35/F	RA (5 y), BCC (6 m)	86	1/320	640	ND	Pos	ND	Yes	Corticosteroids
12	50/F	BCC (6 m)	113	1/80	29	Pos/Neg	Neg	Pos	Yes	MTX
13	28/F	BCC (10 y), AITD	103	Neg	ND	ND	ND	ND	Yes	None
14	47/F	BCC (1 y), AITD	130	ND	ND	ND	ND	ND	No	Corticosteroids/MTX
15	55/F	BCC (1 y), glomerulonephritis (10 y)	200	ND	ND	ND	ND	ND	Yes	MTX/Corticosteroids
16	52/F	BCC (2 y)	115	Neg	Neg	Neg	ND	ND	Yes	CSA/G-CSF
17	54/M	BCC (3 y)	145	Neg	Neg	Neg	ND	ND	No	Unknown
18	70/M	BCC (1 y), weight loss, skin lesions	115	ND	ND	ND	ND	ND	No	MTX/Corticosteroids
19	66/F	BCC, recurrent infections, weight loss (10 y)	120	Neg	Neg	ND	ND	ND	Yes	Unknown
20	69/F	BCC (6 y)	125	Neg	Neg	ND	ND	ND	Yes	Unknown
21	33/F	BCC (6 m)	150	1/80	160	ND	ND	ND	Yes	CSA

aCL, anticardiolipin antibody; AITD, autoimmune thyroiditis; ANA, antinuclear antibodies; ANCA, antineutrophil cytoplasmic antibodies; BCC, blood cell count abnormalities; CCP, anticyclic citrullinated peptide antibodies; CSA, cyclosporine A; dsDNA, double-stranded DNA; F, female; G-CSF, granulocyte-colony stimulating factor; HP, polyclonal hipergammaglobulinemia; m, months of observation; M, male; MTX, methotrexate; ND, not done; Neg, negative; Pos, positive; RA, rheumatoid arthritis; RF, rheumatoid factor; UA, unclassified arthritis; y, years of observation.

**Table 2:** Hematological data and T-cell receptor and immunoglobulin gene rearrangements

Case	Hemoglobin, g/dL	WBC, × 10^9^/L	Absolute neutrophil count, × 10^9^/L	Absolute lymphocyte count, × 10^9^/L	Absolute LGL count, × 10^9^/L	Platelet count, × 10^9^/L	*TCR *and *IG *gene rearrangements
1	14.1	2.4	0.31	2.0	1.4	204	V_β_-J_β2_, V_γ _If, V_γ10_-J_γ_, V_γ9_, V_γ11_-J_γ_
2	14.6	7.6	0.22	7.1	4.6	148	V_β_-J_β1_, J_β2_, V_β_-J_β2_, V_γ _If, V_γ__10_-J_γ_
3	11.3	1.6	0.05	1.3	0.9	103	ND
4	8.2	15.7	1.45	12.9	11.4	381	V_β_-J_β1_, J_β2_, V_β_-J_β2_, D_β1_, D_β2_-J_β_, V_γ _If, V_γ10_-J_γ_, V_γ9_, V_γ11_-J_γ _V_κ_-J_κ_
5	13.4	9.2	1.5	6.6	4.1	230	D_β1_, D_β2_-J_β_, V_γ _If, V_γ10_-J_γ_, V_δ_, D_δ2_-J_δ_, D_δ3 _(biclonal)
6	10.6	2.4	0.31	1.6	1.1	234	V_β_-J_β1_, J_β2_, D_β1_, D_β2_-J_β_, V_γ _If, V_γ10_-J_γ_
7	14.1	1.1	0.15	0.8	0.75	113	V_γ _If, V_γ10_-J_γ_, V_γ9_, V_γ11_-J_γ_, V_δ_, D_δ2_-J_δ_, D_δ3 _V_κ_, intron-Kde; V_λ_-J_λ_
8	11.4	2.6	0.39	1.8	0.9	100	V_β_-J_β1_, J_β2_, D_β1_, D_β2_-J_β_
9	11.9	0.9	0.09	0.8	0.5	98	V_β_-J_β1_, J_β2_, V_β_-J_β2 _V_κ_-J_κ_; V_κ_, intron-Kde (biclonal or biallelic); V_λ_-J_λ_
10	12.1	1.3	0.14	0.8	0.3	112	No rearrangement
11	10.8	1.2	0.06	1.02	0.4	321	No rearrangement
12	9.7	8.7	0.12	7.9	7.1	192	V_β_-J_β1_, J_β2_, D_β1_D_β2_-J_β _(biclonal or biallelic), V_γ _If V_γ10_-J_γ_
13	12.2	8.52	0.72	6.9	6.5	301	V_β_-J_β1_, J_β2_, D_β1_, D_β2_-J_β_, V_γ _If, V_γ10_-J_γ _(biclonal or bliallelic)
14	7.8	7.7	0.8	6.3	4.5	21	ND
15	10.9	17.8	0.71	16.4	11.8	285	V_β_-J_β1_, J_β2_, D_β1_, D_β2_-J_β _(biclonal or biallelic), V_γ _If, V_γ10_-J_γ_, V_γ9_, V_γ11_-J_γ _(biclonal or biallelic) V_κ_-J_κ_
16	13.0	8.9	0.35	8.5	4.6	200	V_β_-J_β1_, J_β2_, V_β_-J_β2_, D_β1_, D_β2_-J_β_, V_γ _If, V_γ10_-J_γ_, V_γ9_, V_γ11_-J_γ_
17	14.4	2.65	0.10	2.4	2.0	115	D_β1_, D_β2_-J_β_
18	14.9	36.4	0.66	34.5	26.9	57	V_β_-J_β1_, J_β2_, V_β_-J_β2_, D_β1_, D_β2_-J_β_, V_γ9_, V_γ11_-J_γ_, V_δ_, D_δ2_-J_δ_, D_δ3 _(biclonal or biallelic)
19	10.2	9.6	0.85	8.4	5.7	154	V_β_-J_β1_, J_β2_, V_β_-J_β2_, V_γ _If, V_γ10_-J_γ _V_γ9_, V_γ11_-J_γ_, V_δ_, D_δ2_-J_δ_, D_δ3_
20	9.5	8.5	0.52	7.2	5.4	220	V_β_-J_β1_, J_β2_, V_β_-J_β2_, V_γ _If, V_γ10_-J_γ_
21	4.5	4.5	0.74	3.7	2.1	228	V_β_-J_β1_, J_β2_, (biclonal or biallelic), V_β_-J_β2 _V_γ _If, V_γ10_-J_γ _(biclonal or biallelic)

IG, immunoglobulin; LGL, large granular lymphocyte; ND, not done; TCR, T-cell receptor; WBC, white blood cells.

Eleven patients with articular disease demonstrated various degrees of inflammatory arthropathy. Nine patients had long-lasting (5 to 43 years) RA with erosions and fulfilled the ARA diagnosis criteria. RA preceded the onset of hematological abnormalities by 3 to 43 years. All of these patients had positive RF (RF-IgM), CCP antibodies, and ANAs as well as polyclonal hypergammaglobulinemia and were diagnosed as FS due to neutropenia and/or splenomegaly [18]. Two patients (8 and 9) had unclassified arthritis that resembled RA but did not fulfill the ARA criteria for this diagnosis. Their articular disease was symmetrical and peripheral with arthralgia, stiffness, periodic swelling, and subchondral cysts on ultrasonography, but no erosions. In both patients, aCLs were detected. ANAs were positive in patient 8, and antibodies to double-stranded DNA, RF-IgM, and anti-CCP were positive in patient 9. Both patients presented with splenomegaly, recurrent infections due to severe neutropenia, and skin lesions. In one (patient 8), arthropathy was observed 7 years before hematological abnormalities whereas in the other (patient 9) it appeared after a 17-year history of leucopenia, LGL lymphocytosis, and neutropenia. In 10 patients, there were no symptoms of arthritis or serological abnormalities except polyclonal hypergammaglobulinemia in 7 patients, aCL in 1 patient, and RF in 1 patient. Cytoplasmic antineutrophil antibodies were positive in 1 of 10 tested patients (patient 12).

Four patients had constitutional symptoms such as fatigue and weight loss. Ten patients demonstrated splenomegaly (>14 cm splenic axis in ultrasonography). Three had recurrent bacterial infections of the respiratory tract (sinusitis, bronchitis, and pneumonia) and 1 patient had a foot abscess. In 3 patients, skin lesions in the form of macular pigmented skin rash were observed. Autoimmune thyroiditis was documented in 5 patients.

### Bone marrow morphology and immunohistochemistry

**Figure 1:**
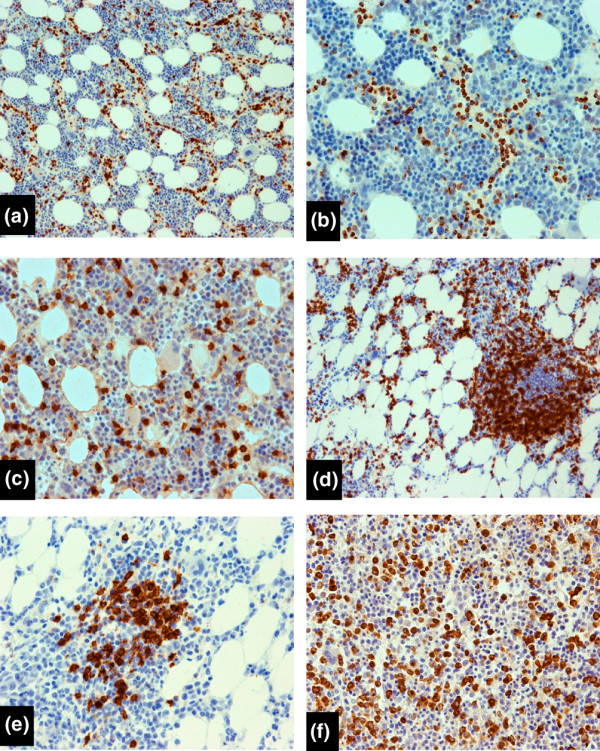
Histopathological features of bone marrow in patients with arthritis and T-cell large granular lymphocyte (T-LGL) lymphocytosis. **(a) **Patient 1 with rheumatoid arthritis (RA) and T-LGL leukemia. Staining for CD57 demonstrates intrasinusoidal linear arrays and interstitial clusters of T cells (EnVision stain, ×100). **(b) **Granzyme B highlights cytotoxic granules in these cells (EnVision stain, ×200). **(c) **Patient 10 with polyclonal T-LGL lymphocytosis. Staining for CD8 shows dispersed T cells (EnVision stain, ×200). **(d) **Patient 9 with unclassified arthritis, T-LGL leukemia, and *IGKV *and *IGLV *gene rearrangements. CD3 staining shows interstitial and nodular infiltration of T cells (EnVision stain, ×100). **(e) **Patient 9. The lymphoid nodule contains few CD20^+ ^B cells (EnVision stain, ×200). **(f) **Patient 7 with RA and T-LGL leukemia. A decreased count of granulocytic precursors (myeloperoxydase^+^) is shown (EnVision stain, ×200). IGKV, immunoglobulin kappa variable; IGLV, immunoglobulin lambda variable

Morphological and immunohistochemical bone marrow characteristics are summarized in Table 3. The bone marrow was hypercellular in 11 patients, normocellular in 5 patients, and hypocellular in 5 patients. Sections stained with monoclonal antibodies revealed interstitial infiltrates of small lymphocytes with slightly irregular nuclei and scanty cytoplasm, which formed small clusters and aggregates in all patients with *TCR *gene rearrangements. Moreover, in 14 of them, the infiltrates also had a clear intrasinusoidal linear component (Figure 1a). These infiltrations were subtle and difficult to notice on standard hematoxylin and eosin stain. T cells were CD3^+^, CD8^+^, granzyme B^+^, and CD4^- ^in 16 patients (Figure 1b). Three patients had different phenotypes of T cells: CD3^+^/CD4^-^/CD8^-^, CD3^+^/CD4^+^/CD8^-^, and CD3^+^/CD4^+^/CD8^+^. CD57 staining gave variable results and was positive in 12 patients, positive in only some T cells in 5 patients, and negative in 2 patients. In two cases (patients 10 and 11) with polyclonal T-LGL lymphocytosis, CD3^+^CD8^+^CD57^+/-^/granzyme B^+/- ^lymphocytes were dispersed in the bone marrow and did not form clusters or intravascular infiltrations (Figure 1c). Reactive intertrabecular lymphoid nodules were detected in 14 of 21 examined patients (Figure 1d). B cells in the center of these nodules expressed CD20 and, in 2 cases, formed germinal centers (Figure 1e). They were surrounded by small CD3^+ ^T lymphocytes expressing predominantly CD4^+ ^and only a few CD8^+ ^cells. Myeloperoxydase stain showed decreased granulocyte precursors with left-shifted maturation in 12 patients, normal in 7 patients, and increased in 2 patients (Figure 1f). Red cell precursors revealed normal maturation. In most cases, the megakaryocyte count and their morphology were normal.

**Table 3:** Bone marrow morphological and immunophenotypic characteristics

Number	Cellularity	Type of infiltrate	Percentage of LGLs	Phenotype by IHC	Phenotype by flow cytometry	RCP	GP	Meg	LN
1	N	IC, IVL	35	3^+^, 4^-^, 8^+^, 57^+^, gr B^+^	2^+^, 3^+^, 4^-^, 5^+^, 7^+/-^↓, 8^+^, 16^+/-^, 56^-^, 57^+^	N	D, LSM	N	+
2	N	IC, IVL	30	3^+^, 4^-^, 8^+^, 57^+^, gr B^+/-^	ND	N	I, LSM	N	+
3	N	IC	50	3^+^, 4^-^, 8^+^, 57^+/-^, gr B^+^	2^+^, 3^+^, 4^-^, 5^+/-^↓, 7^+^, 8^+^, 16^+/-^, 56^-^, 57^+^, TCRαβ^+^, TCRγδ^-^	N	D, LSM	N	-
4	I	IC, IVL	58	ND	2^+^, 3^+^, 4^-^, 5^-^, 7^+/-^↓, 8^+^,16^+/-^, 56^-^, 57^-^, 43^+^↓, 25^-^, TCRαβ^+^, TCRγδ^-^, HLA-DR^+/-^	D	N, LSM	N	+
5^a^	D	IC, IVL	15	3^+^, 4^-^, 8^-^, 57^+^, gr B^+/-^	2^+^, 3^+^, 4^-^, 5^+/-^↓, 7^+^, 8^-^, 16^+/-^, 56^+/-^, 57^-^, 43^+^, 25^-^, TCRαβ^-^, TCRγδ^+^, HLA-DR^+ ^and 2^+^, 3^+^, 4^-^, 5^-^, 7^+^, 8^+/-^, 16^+^, 56^+/-^, 57^+^, 43^+^, 25^-^, TCRαβ^-^, TCRγδ^+^, HLA-DR^+^	N	N, LSM	N	+
6	I	IC	40	3^+^, 4^-^, 8^+^, 57^+^, gr B^+^	ND	N	D, LSM	N	+
7	I	IC	80	3^+^, 4^-^, 8^-/+^, 57^+/-^, gr B^+^	2^+^, 3^+^, 4^-^, 5^+^, 7^+/-^↓, 8^+/-^, 16^+/-^, 56^+/-^, 57^+^, TCRαβ^-^, TCRγδ^+^, HLA-DR^+/-^	N	D, LSM	N	-
8	N	IC, IVL	25	3^+^, 4^+^, 8^-^, 57^+^, gr B^+^	ND	N	D, LSM	N	+
9	I	IC, IVL	35	3^+^, 4^+^, 8^+^, 57^+^, gr B^+/-^	ND	N	D, LSM	D	+
10	I	DI	15	3^+^, 4^-^, 8^+^, 57^+/-^, gr B^+/-^	2^+^, 3^+^, 4^-^, 5^+^, 7^+^, 8^+^, 16^+/-^, 56^+/-^, 57^+/-^, HLA-DR^-/+^	N	I, LSM	N	+
11	D	DI	15	3^+^, 4^-^, 8^+^, 57^+/- ^gr B^+/-^	2^+^, 3^+^, 4^-^, 5^+^, 7^+^, 8^+^, 16^+/-^, 56^-^, 57^+/-^	N	D, LSM	N	-
12	I	IC, IVL	50	3^+^, 4^-^, 8^+^, 57^+^, gr B^+^	2^+^, 3^+^, 4^-^, 5^+/-^↓, 7^+^, 8^+^, 16^+^, 56^+/-^, 57^+/-^, 25^-^, HLA-DR^+/-^	N	D, LSM	N	+
13	D	IC, IVL	20	3^+^, 4^-^, 8^+^, 57^+^, gr B^+^	2^+^, 3^+^, 4^-^, 5^+^, 7^-^↓, 8^+^, 16^+^, 56^-^, 57^+/-^, 43^+^↓, 25^-^, TCRαβ^+^, TCRγδ^-^, HLA-DR^+^	D	N, LSM	N	-
14	I	IC	20	3^+^, 4^-^, 8^+^, 57^-^, gr B^+/-^	2^+^, 3^+^, 4^-^, 5^+^, 7^+/-^↓, 8^+^, 16^+/-^, 56^-^, 57^-^, 25^-^, TCRαβ^+^, TCRγδ^-^, HLA-DR^+/-^	D	N, LSM	N	-
15	I	IC, IVL	20	3^+^, 4^-^, 8^+^, 57^-^, gr B^+^	2^+^, 3^+^, 4^-^, 5^-^↓, 7^-^↓, 8^+^, 16^+/-^, 56^-^, 57^-^, 43^+^↓, 25^-^, TCRαβ^+^, TCRγδ^-^, HLA-DR^+/-^	N	D, LSM	N	+
16	N	IC, IVL	25	3^+^, 4^-^, 8^+^, 57^+^, gr B^+/-^	2^+^, 3^+^, 4^-^, 5^-^↓, 7^-^↓, 8^+^, 16^+/-^, 56^-^, TCRαβ^+^, TCRγδ^-^, HLA-DR^+^	N	D, LSM	N	+
17	D	IC, IVL	60	3^+^, 4^-^, 8^+^, 57^+^, gr B^+^	2^+^, 3^+^, 4^-^, 5^-^↓, 7^-^↓, 8^+^	N	D, LSM	N	-
18	I	IC, IVL	25	3^+^, 4^-^, 8^+^, 57^+/-^, gr B^+^	2^+^, 3^+/-^↓, 4^-^, 5^+/-^↓, 7^+/-^↓, 8^+^, 16^+^, 56^-^, 57^+/-^, 43^+^↓, 25^-^, TCRαβ^+^, TCRγδ^-^, HLA-DR^+^	N	N, LSM	N	-
19	I	IC	30	3^+^, 4^-^, 8^+^, 57^+/-^, gr B^+^	2^+^, 3^+^, 4^-^, 5^+^, 7^+/-^↓, 8^+^, 16^+^, 56^-^, 57^+/-^, 25^-^, TCRαβ^+^, TCRγδ^-^, HLA-DR^+^	D	N, LSM	D	+
20	D	IC, IVL	25	3^+^, 4^-^, 8^+^, 57^+^, gr B^+^	ND	D	N, LSM	N	+
21	I	IC, IVL	25	3^+^, 4^-^, 8^+^, 57^+^, gr B^+^	ND	N	D, LSM	N	+

^a^Two cell populations.

Expression of antigens: -, lack of antigen expression; +/-, partial expression varying from 20% to 95% of cells; +, expression of antigens on 100% of cells; ↓, abnormal expression (dim, partial, or negative expression) and CD43 (weaker level). D, decreased; DI, dispersed; GP, granulocyte precursors; gr B, granzyme B; I, increased; IC, interstitial clusters; IHC, immunohistochemistry (phenotype in expression of CD markers); IVL, intravascular lymphocytes; LGLs, large granular lymphocytes; LN, lymphoid nodules; LSM, left-shifted maturation; Meg, megakaryocyte; N, normal; ND, not done; RCP, red cell precursors; TCR, T-cell receptor.

### Flow cytometry immunophenotyping

**Figure 2:**
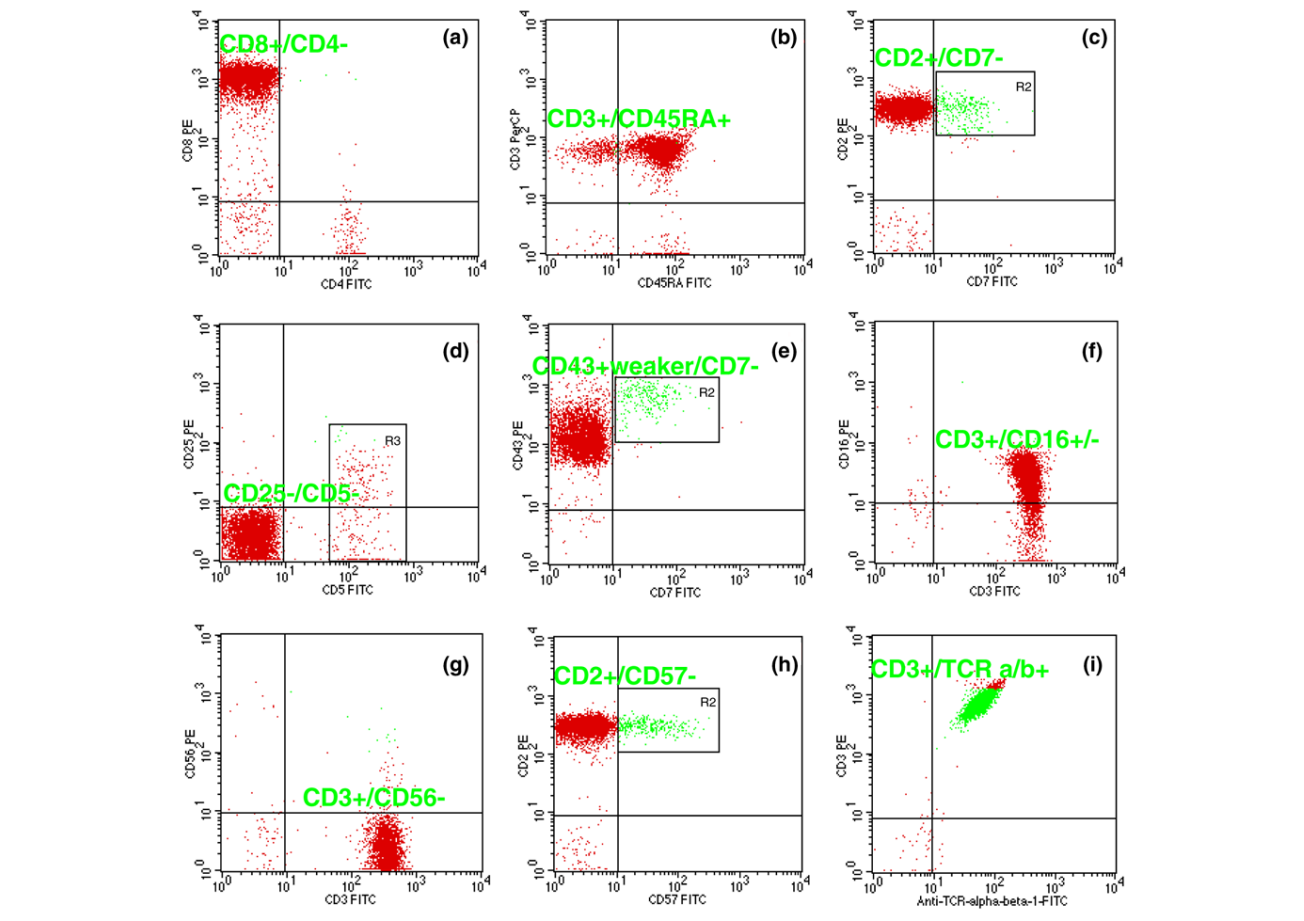
Flow cytometric analyses of patient 15 with T-cell large granular lymphocyte leukemia. **(a) **CD8^+^CD4^- ^leukemic cells. **(b) **CD3^+^/CD45RA^+ ^leukemic cells. **(c) **CD2^+^/CD7^- ^leukemic cells and double-stained population in the region R2 consistent with normal T lymphocytes. **(d) **CD5^-^/CD25^- ^leukemic cells and CD5^+^/CD25^-/+ ^expression on normal T lymphocytes in the region R3. **(e) **CD7^- ^leukemia cells express slightly weaker levels of CD43 compared with normal CD43^+higher^CD7^+ ^cells consistent with normal T lymphocytes in the region R2. **(f) **CD3^+ ^population with coexistence of CD16 antigens. **(g) **CD3^+^CD56^- ^leukemic cells. **(h) **CD2^+^CD57^- ^leukemic cells. CD2^+^CD57^+^-reactive T lymphocytes in the region R2. **(i) **Leukemic cells are positive for CD3 and TCRαβ. FITC, fluorescein isothiocyanate; PE, phycoerythrin; TCR, T-cell receptor.

The results of lymphocyte surface marker analysis performed in 15 patients are summarized in Table 3. The typical immunophenotype of T-LGL leukemia cells was CD45^+bright^, CD2^+bright^, CD3^+bright^, CD4^-^, CD8^+bright^, CD25^-^, and CD43^+weaker^. CD5 and CD7 expression was variable (bright, dim, or negative) on all or part of the T-LGL leukemia cells, whereas in 3 cases lymphocytes showed an absence of both antigens. In all studied cases, T-LGL leukemia cells expressed a slightly weaker level of CD43 as compared with normal expression of CD43^+higher ^on T lymphocytes. Aberrant expression of CD3 was found in only 1 patient. All tested cases expressed CD16. However, 10 cases showed only partial expression of this antigen, with 20% to 95% of the T-LGL leukemia cells showing reactivity. Lack of CD56 expression was noted in 10 cases; in 2 cases, CD56 was expressed in more than 50% of the T-LGL leukemia cells. In 10 cases, 20% to 100% of the T-LGL leukemia cells showed expression of CD57, whereas 3 cases were negative. HLA-DR was expressed in all tested cases in varying percentages. TCR proteins were tested in 10 cases, 8 of them expressing TCRαβ and 2 TCRγδ (Figure 2). Patient 5 with TCRγδ protein expression had two immunophenotypically different populations of T-LGL leukemia cells and is the subject of a separate report. In analyzed cases both normal reactive peripheral blood CD57^+ ^T lymphocytes and CD57^+ ^T-LGL leukemia cells were found. In the former no loss of expression of any pan-T antigens was observed, in the latter typical abnormalities of pan-T-cell antigens were noted. In patients 10 and 11, with no rearrangement of the *TCR *genes, T-LGLs were characterized by normal expression of T-cell antigens.

### Genetic analysis

**Figure 3:**
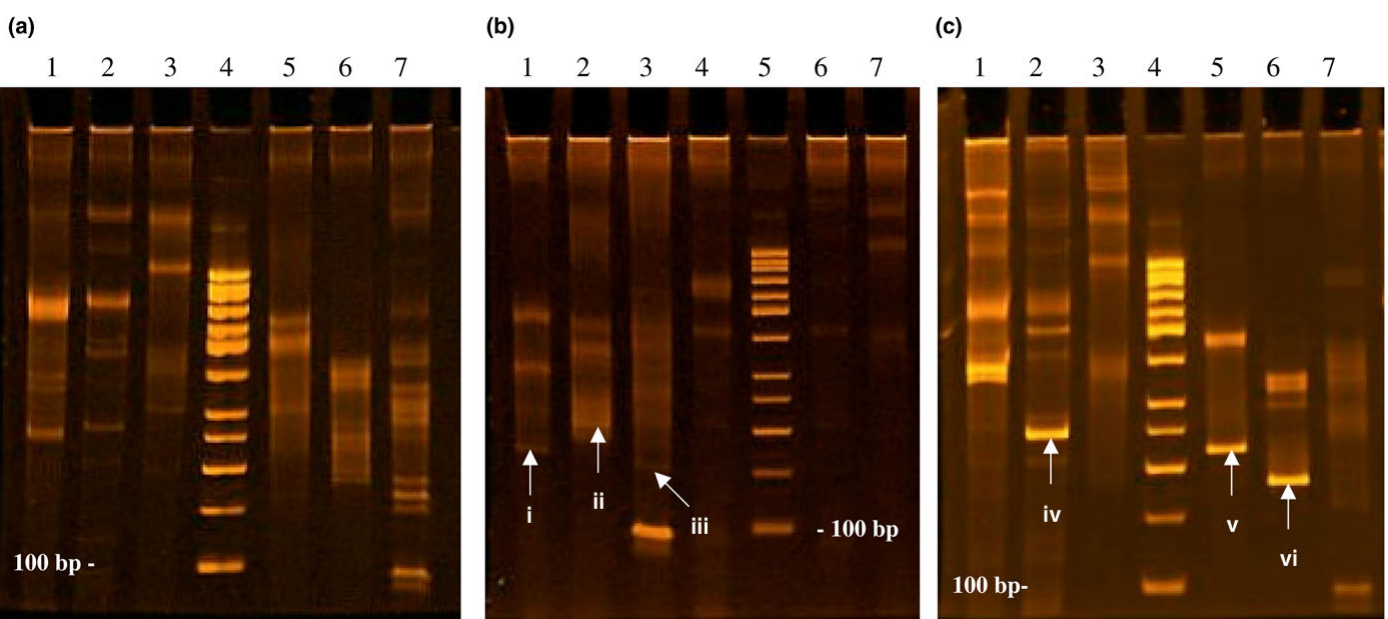
Ethidium bromide-stained polyacrylamide gel showing polymerase chain reaction products derived from *TCR *gene rearrangements in patients with rheumatoid arthritis and T-cell large granular lymphocyte (T-LGL) proliferations. **(a) **Polyclonal expansion of T-LGLs in patient 10. Lane 1: *TCRB *gene rearrangement–negative, polyclonal smear (tube A); lane 2: *TCRB *gene rearrangement-negative, polyclonal smear (tube B); lane 3: *TCRB *gene rearrangement-negative, polyclonal smear (tube C); lane 4: standard 50 base pairs (bp); lane 5: *TCRG *gene rearrangement-negative, polyclonal smear (tube A); lane 6: *TCRG *gene rearrangement-negative, polyclonal smear (tube B); and lane 7: *TCRD *gene rearrangement-negative, polyclonal smear. **(b) **Monoclonal expansion in polyclonal background in patient 7. Lane 1: *TCRG *gene rearrangement: monoclonal product 180 bp (i) in tube A; lane 2: *TCRG *gene rearrangement: monoclonal product 210 bp (ii) in polyclonal background (tube B); lane 3: *TCRD *gene rearrangement: monoclonal product 160 bp (iii); lane 4: *TCRB *(tube A) gene rearrangement-negative, polyclonal smear; lane 5: standard 50 bp; lane 6: *TCRB *(tube B) gene rearrangement-negative, polyclonal smear; and lane 7: *TCRB *(tube C) gene rearrangement-negative, polyclonal smear. **(c) **Monoclonal gene rearrangements in patient 1 with T-LGL leukemia. Lane 1: *TCRB *gene rearrangement-negative (tube A); lane 2: *TCRB *gene rearrangement-positive, monoclonal product 250 bp (iv) in tube B; lane 3: *TCRB *(tube C): gene rearrangement-negative, polyclonal smear; lane 4: standard 50 bp; lane 5: *TCRG *gene rearrangement-positive, monoclonal product 230 bp (v) in tube A; lane 6: *TCRG *gene rearrangement-positive, monoclonal product 180 bp (vi) in tube B; and lane 7: *TCRD *gene rearrangement-negative, polyclonal smear. TCR, T-cell receptor.

The detailed results of *TCR *gene rearrangement tests are summarized in Table 2. Two patients (patients 10 and 11) had no rearrangement in *TCR *genes consistent with polyclonal lymphocytosis (Figure 3a). Patient 7, apart from monoclonal *TCR *rearrangement in the delta chain, showed a weak monoclonal product in the *TCRG *V_γ9_, V_γ11_-J_γ _region in polyclonal background (Figure 3b). Clearly monoclonal *TCR *gene rearrangements were detected in 16 patients (Figure 3c). Three patients had rearrangements in genes coding beta chains, 10 showed clonality in genes coding beta and gamma chains, and 3 had rearrangement in genes coding beta, gamma, and delta chains. The spectrum of *TCR *gene rearrangements was quite variable, although there were some repeated uses observed. The most frequently rearranged regions of variable genes were V_β_-J_β1_, J_β2 _(13 patients) and V_γ _If V_γ10_-J_γ _(13 patients). Moreover, 4 patients (patients 4, 7, 9, and 15) also presented rearrangements of immunoglobulin kappa variable (*IGKV*) or lambda variable (*IGLV*) genes. Classic cytogenetic tests with GTG banding of the bone marrow cells were performed in 7 patients (patients 1, 4, 9, 12, 13, 15, and 18) and revealed a normal karyotype.

### Therapy and follow-up

Various therapeutic approaches were used as presented in Table 1. The T-LGL leukemia treatment corrected cytopenias: neutropenia in 14 and anemia in 4 patients as well as symptoms of arthritis. Therapy included methotrexate (MTX), cyclosporine A (CSA), corticosteroids, and granulocyte-colony stimulating factor (G-CSF). The overall response rate to therapy in our series was 50%. None of the treated patients achieved complete hematologic remission. In 8 patients with T-LGL leukemia, low oral doses of MTX (7.5 to 15 mg weekly) for 1 to 42 months (median duration 14.4 months) were given. Six patients received concomitant prednisone (5 to 40 mg daily) or G-CSF. In 5 patients (patients 4, 9, 12, 15, and 18), the response was partial (PR), which was defined as improvement of the blood cell count by more than 50%. Two patients received oral CSA therapy (2 and 3 mg/kg daily) for 15 and 21 months, and, in both, PR was achieved. One of them received a combination of CSA and G-CSF therapy. Prednisone alone (5 to 10 mg daily) was given to 4 patients for 12 to 34 months (median duration 20 months) as a continuation of previous arthritis treatment. No response was noted in these patients, but anemia and recurrent infections as well as RA were controlled. In all of the patients, arthritis responded well to the treatment. Two patients with FS and polyclonal T-LGL lymphocytosis were treated with a combination of MTX, CSA, and prednisone as well as prednisone alone. The first patient achieved PR, and the second achieved only a transient improvement of neutropenia. During 1 to 17 years of follow-up, hematologic disease remained indolent in the majority of patients, with the exception of 2 patients. Patient 14 died due to severe bacterial pneumonia complicated by sepsis, disseminated intravascular coagulation, and myocardial infarction. An autopsy was not performed but death most probably was related to T-LGL leukemia-associated neutropenia. Patient 2 died of a cause unrelated to disease: secondary renal amyloidosis (Amyloid A), a complication of long-lasting RA.

## Discussion

The pathogenic relationship between RA and various T-LGL proliferations that are a spectrum of disorders from reactive expansion of 'normal' CD8^+ ^cytotoxic T lymphocytes through chronic oligoclonal or monoclonal LGL lymphocytosis to clinically overt T-LGL leukemia is unclear [4]. This association is considered rare, perhaps due to underdiagnosis of T-LGL proliferations as the cause of neutropenia in patients with RA [3]. We have evaluated data of 21 T-LGL lymphocytosis patients and identified a relatively high proportion of patients with arthropathy, also reported by some authors [3,10,19,20]. Nine patients had RA and neutropenia and some also had splenomegaly and were diagnosed as FS, whereas 2 other patients showed milder non-erosive unclassified arthritis. Our FS patients presented a spectrum of T-LGL expansions.

It is difficult to differentiate between T-LGL leukemia and reactive T-LGL proliferations, especially in autoimmune diseases. Molecular studies may establish the clonal nature of T cells but not in all cases confirm the diagnosis of leukemia as oligoclonal/monoclonal proliferations of T-LGL may occur in natural or pathologic immune responses to strong antigens in autoimmune diseases or viral infections such as Epstein-Barr virus and HIV [21]. A monoclonal population of CD8^+^, CD57^+ ^T cells was found both in neutropenic and non-neutropenic patients with RA as well as in healthy, mostly elderly individuals [11,22-24]. All these cases represent a benign monoclonal T-LGL proliferation rather than true T-LGL leukemia because they are clinically asymptomatic. Moreover, diagnostic criteria of T-LGL leukemia in the context of the LGL population volume are still a subject of discussion. Monoclonal LGL lymphocytosis of greater than 2 to 5 × 10^9^/L is required for the diagnosis of T-LGL leukemia according to the WHO classification [6]. However, in patients with monoclonal T-LGL lymphocytosis and leucopenia, this criterion is not useful. Expansion of LGLs of less than 0.5 × 10^9^/L represents reactive lymphocytosis [6], but there are also borderline cases with T-LGL oligoclonal or monoclonal lymphocytosis values ranging from 0.5 to 2 × 10^9^/L. For such cases, Dhodapkar and colleagues [10] suggested the term 'T cell clonopathy of undetermined significance' or 'monoclonal clonopathy of unclear significance'. However, Semenzato and colleagues [25] described 9 patients with chronic monoclonal T-LGL lymphocytosis of 0.5 to 2 × 10^9^/L with clinical and laboratory features typical for T-LGL leukemia. Thus, they pointed out that, for T-LGL leukemia diagnosis, the LGL count by itself is not critical but a comprehensive analysis of clinical, immunopathological, and molecular data is necessary. However, the border between leukemic and reactive clonal expansions of T-LGLs is narrow because clinical and hematologic characteristics of T-LGL leukemia are far from typically malignant [9].

In our group of patients with arthritis, 9 presented symptoms of FS. Three patients (patients 2, 4, and 5) fulfilled the WHO criteria for T-LGL leukemia diagnosis with absolute LGL lymphocytosis of greater than 2 × 10^9^/L and monoclonal rearrangement of *TCRB*, *TCRG*, or *TCRD *genes. In the bone marrow, they had interstitial and intrasinusoidal linear infiltration of T cells expressing CD3, CD8, CD57, and granzyme B described by Morice and colleagues [26] as a typical pattern for T-LGL leukemia. Intrasinusoidal infiltration seems to indicate a leukemic nature of the disease and may occur in other subtypes of leukemia/lymphoma [27]. Reactive lymphoid nodules, reported by Osuji and colleagues [28] to be frequent in T-LGL leukemia, were also present. In FCM, T-LGL leukemia cells revealed abnormal expression of CD5, CD7, and CD43 antigen, which corresponds to the findings of Lundell and colleagues [29]. The other 6 patients with FS showed relative LGL lymphocytosis but had absolute LGL lymphocytosis of less than 2 × 10^9^/L due to leucopenia.

In three of those 6 patients (patients 1, 3, and 6) with LGL lymphocytosis ranging from 0.9 to 1.4 × 10^9^/L, a clearly monoclonal rearrangement of *TCRB *and *TCRG *genes as well as typical for T-LGL leukemia immunophenotype were found. However, interstitial infiltration of the bone marrow was most common. In one patient, FCM showed abnormal expression of one T-cell antigen. The question is whether these cases should be diagnosed as true T-LGL leukemia or as a T-cell clonopathy of unclear significance associated with FS.

Our patient 7 displayed features of monoclonal transformation of polyclonal LGLs. In PCR analysis, apart from monoclonal *TCRD *gene rearrangement, a weak monoclonal product in the V_γ9_, V_γ11_-J_γ _region among polyclonal background was found. Abundant interstitial infiltrates of T cells were observed in the bone marrow, but without intrasinusoidal localization. This case seems to support the hypothesis that T-LGL leukemia may develop from polyclonal or oligoclonal expansions [30] and correlates with the report of Langerak and colleagues [31], who found a single dominant and several weak additional gene products in β-chain variable region (V_β_) transcripts in numerous T-LGL leukemia cases.

Two patients (patients 10 and 11) with no rearrangement of *TCR *genes may serve as examples of autoimmune-disease-related, polyclonal, reactive, chronic proliferation of LGL lymphocytes. In these two cases, the pattern of bone marrow infiltration and FCM analysis were consistent with reactive T-LGL lymphocytosis [26,29]. T-LGLs were dispersed in the bone marrow and they did not lose any pan-T-cell antigens in FCM.

As in other reports [29], FCM analysis of all of our cases with clonal rearrangement of *TCR *genes showed normal reactive peripheral blood CD57^+ ^T lymphocytes with no pan-T-cell antigen abnormalities in addition to a population of CD57^+ ^T-LGL leukemia cells. It is conceivable that this population of cells may represent precursors of T-LGL leukemia.

The clinical course of 9 patients with FS, irrespective of T-LGL absolute count and their clonality, was similar, with neutropenia being the most common presentation, and remained indolent for 1 to 7 years of follow-up. They were treated with immunomodulatory agents exclusively, and 2 patients had partial hematologic responses.

The described group of patients represents a spectrum of T-LGL proliferations and supports the hypothesis that monoclonal expansion of LGLs with features of T-LGL leukemia may result from the transformation of initially stable and benign polyclonal or oligoclonal proliferation of these lymphocytes [30]. This proliferation may be a consequence of the disregulated reaction of the immune system to viral or auto-antigens associated with autoimmune processes [30,32]. There are similarities in immunogenetic profile between T-LGL leukemia and autoimmune diseases [32]. Moreover, molecular analysis revealed common motifs in the *TCRB *genes in T-LGL proliferations, suggesting a potential role of antigenic stimulation in the clonal evolution of the disease [33-35]. Unfortunately, the triggering antigens and genetic events that cause neoplastic transformation remain unknown. Bowman and colleagues [11] examined two groups of patients with FS, without and with clonal proliferation of T-LGLs, but no continuous distributions of T-LGLs in these two groups were observed. Recently, Langerak and colleagues [4] and Sandberg and colleagues [36] have shown a continuous spectrum of T-LGL proliferations using sensitive PCR techniques.

It is worth emphasizing that, in 4 of our patients, *IGKV *and *IGLV *gene rearrangements were detected in PCR analysis. Trephine biopsy examination showed a nearly normal number of B lymphocytes dispersed and/or localized in lymphoid nodules with the pattern and phenotype indicative of their reactive nature. This phenomenon most probably is due to chronic antigen stimulation in autoimmune disease [37]. It can also correspond to crosslineage light-chain gene rearrangements. None of our patients developed a B-cell lymphoma during follow-up.

Most of our patients had long-lasting RA at the time of diagnosis of T-LGL leukemia, but 2 patients showed milder non-erosive unclassified arthritis resembling RA. Similar findings had been reported by Snowden and colleagues [3]. Interestingly, these 2 patients had similar clinical features: T-LGL absolute count of less than 2 × 10^9^/L due to leucopenia, severe neutropenia, recurrent infections, skin lesions, splenomegaly, and monoclonal rearrangements of *TCRB *genes. Bone marrow infiltration was typical for T-LGL leukemia, but T-LGL cells had the unusual phenotype (CD4^+^). In one of these patients, arthropathy appeared after 17 years of hematological abnormalities, which indicates that T-LGL expansion may also precede inflammatory arthropathy and may be responsible for the development of immunological abnormalities [8]. TCRαβ^+^/CD4^+ ^T-LGL lymphocytosis is a rare subgroup of monoclonal LGL lymphoproliferations and usually is characteristic of a more indolent clinical course [35].

Patients with T-LGL leukemia with or without arthropathy share many similarities. Arthritis and T-LGL leukemia patients more often had leucopenia and splenomegaly and a higher incidence of specific autoantibodies as compared with those without arthropathy. The two groups showed a similar histopathological pattern and immunophenotype of bone marrow infiltration. They also had similar molecular and genetic characteristics with the most frequent rearrangements of V_β_-J_β1_, J_β2 _and V_γ _If V_γ10_-J regions of *TCR *genes and normal karyotype. In other studies, FCM analysis of the V_β _TCR repertoire in T-LGL leukemia cases did not reveal the preferential use of any specific V_β _gene [29,30,38]. Davey and colleagues [20] reported that rearrangement of V_β_-6 genes occurred only in patients with T-LGL leukemias associated with RA, although no unique patterns of junctional sequence rearrangement were seen for patients with T-LGL leukemia with and without arthritis.

Cytopenias (especially neutropenia) that appear in the course of RA-associated T-LGL lymphocytosis are the result of humoral and cellular immune mechanisms [7]. Immune complexes or antibodies to neutrophil antigens may induce apoptosis of neutrophils in patients with FS [39]. Compensatory granulocytic hyperplasia with left-shifted maturation is observed in bone marrow as the consequence of excessive peripheral destruction of mature granulocytes [7]. In the described group of patients, only two with FS (one with polyclonal LGL lymphocytosis, the other with T-LGL leukemia) had hypercellular bone marrow with increased granulocyte precursor count and left-shifted maturation, but no antyneutrophil antibodies were present. The majority of our patients with T-LGL leukemia with and without RA showed a decreased or normal number of granulocyte precursor count in the bone marrow with left-shifted maturation. There is a hypothesis that T-LGL leukemia cells may inhibit proliferation and differentiation of granulocytes in the mechanisms of Fas-mediated apoptosis of myeloid precursors and this could be the explanation of granulocyte hypoplasia and paucity of mature forms in the bone marrow [7]. Effector cytotoxic T cells as well as T-LGL leukemia cells express Fas ligand, a member of the tumor necrosis factor family, which can bind Fas and initiate apoptosis in the target cells through this pathway [40]. It is known that progenitor cells (CD34^+^) in the bone marrow may express Fas in response to tumor necrosis factor-alpha (TNF-α) and interferon-gamma (INF-γ) *in vitro *[41] and cytotoxic T lymphocytes in T-LGL leukemia may spontaneously produce INF-γ and TNF-α after stimulation [42].

## Conclusion

RA and neutropenia patients represented a continuous spectrum of T-LGL proliferations, although monoclonal expansions were most frequently observed. Arthritis and T-LGL leukemia patients more often had leucopenia and splenomegaly and a higher incidence of specific autoantibodies as compared with those without arthropathy. The histopathological pattern and immunophenotype of bone marrow infiltration, molecular and genetic characteristics, as well as indolent clinical course were similar in T-LGL leukemia patients, with and without arthritis. Further studies involving patients with RA and T-LGL proliferations may provide important data for a better understanding of RA pathogenesis.

## Abbreviations

aCL = anticardiolipin antibody; ANA = antinuclear antibody; ARA = American Rheumatism Association; BD = Becton Dickinson, San Jose, CA, USA; CCP = anticyclic citrullinated peptide (antibody); CSA = cyclosporine A; ELISA = enzyme-linked immunosorbent assay; FCM = flow cytometry; FS = Felty syndrome; G-CSF = granulocyte-colony stimulating factor; IGH = immunoglobulin heavy chain; IGK = immunoglobulin kappa; IGKV = immunoglobulin kappa variable; IGL = immunoglobulin lambda; IGLV = immunoglobulin lambda variable; INF-γ = interferon-gamma; LGL = large granular lymphocyte; MTX = methotrexate; NK = natural killer; PCR = polymerase chain reaction; PR = partial response; RA = rheumatoid arthritis; RF = rheumatoid factor; TCR = T-cell receptor; T-LGL = T-cell large granular lymphocyte; TNF-α = tumor necrosis factor-alpha; WHO = World Health Organization.

## Competing interests

The authors declare that they have no competing interests.

## Authors' contributions

MP-S conceived of the study, carried out histopathological studies, analyzed data, and wrote the manuscript. GR carried out the flow cytometry studies. MM and HMŁ carried out the molecular genetic studies. KM and RR analyzed clinical data of patients. KW and RM critically reviewed the manuscript. All authors read and approved the final manuscript.
